# A One Health Decalogue for Breastfeeding: Microbiota-Targeted Strategies for Infant Gastrointestinal and Neurodevelopmental Health

**DOI:** 10.3390/nu18071074

**Published:** 2026-03-27

**Authors:** Mariarosaria Matera, Valentina Biagioli, Chiara Maria Palazzi, Martina Meocci, Fausto Pedaci, Alberto Besostri, Nicola Zerbinati, Francesco Di Pierro

**Affiliations:** 1 Microbiota International Clinical Society, 10123 Turin, Italy; 2 Department of Pediatric Emergencies, Misericordia Hospital, 58100 Grosseto, Italy; 3 Department of Neurosciences, Rehabilitation, Ophthalmology, Genetics, Maternal and Child Health, University of Genoa, 16147 Genoa, Italy; 4 Department of Respiratory Medicine, University of Siena, 53100 Siena, Italy; 5 Department of Pediatrics, Valdarno Hospital, 52025 Arezzo, Italy; 6 Preventive and Community Dentistry, School of Dental Hygiene, University of Padova, 35122 Padua, Italy; 7 Department of Medicine and Technological Innovation, University of Insubria, 21100 Varese, Italy; 8 Scientific & Research Department, Velleja Research, 20125 Milan, Italy

**Keywords:** breastfeeding, microbiota, diet, maternal, milk, human, infant, gastrointestinal microbiome, probiotics

## Abstract

**Background/Objectives:** Breastfeeding represents a critical developmental window during which maternal biology, environmental exposures, and nutrition converge to influence infant gastrointestinal health and long-term developmental trajectories. From a One Health perspective, breastfeeding can be conceptualized not as a static nutritional act, but as a dynamic and modifiable biological system in which maternal factors shape early-life microbiota assembly and immune programming. This narrative review explores how microbiota-oriented strategies during breastfeeding may foster a favorable trajectory of infant health, potentially extending to transgenerational outcomes. **Methods:** This narrative review is structured around a ten-point decalogue addressing interconnected domains relevant to the maternal–milk–infant microbiota axis, including maternal diet, microbial diversity, environmental exposures, psychological stress and probiotic use. Current mechanistic and clinical evidence was examined to evaluate how these domains may modulate microbiota composition and function during breastfeeding. Attention was given to probiotic supplementation, including strain specificity, timing of administration, and clinical context, as well as to the broader implications of a One Health framework. **Results:** Available evidence suggests that maternal nutritional patterns, environmental and psychosocial exposures, and targeted microbiota-modulation strategies may influence the composition and functional properties of human milk and the developing infant microbiota. Probiotic use during breastfeeding appears to have strain-specific and context-dependent effects, with potential benefits in selected clinical scenarios. However, findings remain heterogeneous, and uncertainties persist regarding optimal strains, timing, and long-term outcomes. **Conclusions:** Breastfeeding can be understood as a dynamic biological interface shaped by maternal and environmental factors. Integrating microbiota-oriented strategies within a One Health framework may support infant gastrointestinal health and possibly contribute to longer-term developmental trajectories. Nevertheless, careful interpretation of the current evidence is warranted to avoid reductionist, supplement-centered approaches and to prevent maternal overmedicalization or blame.

## 1. Introduction

Early life represents a critical window of biological plasticity during which nutrition, microbial exposures, and environmental signals interact to shape immune, metabolic, and neurodevelopmental trajectories with potential long-term and transgenerational implications [1,2,3]. At the population level, breastfeeding represents one of the most effective early-life health interventions, with strong epidemiological evidence linking optimal breastfeeding practices to reduced infant morbidity and mortality as well as improved long-term health outcomes [1]. Within this window, feeding practices should be interpreted not merely as sources of nutrients, but as complex biological processes capable of modulating host–microbe interactions during a sensitive phase of development.

**Figure 1:**
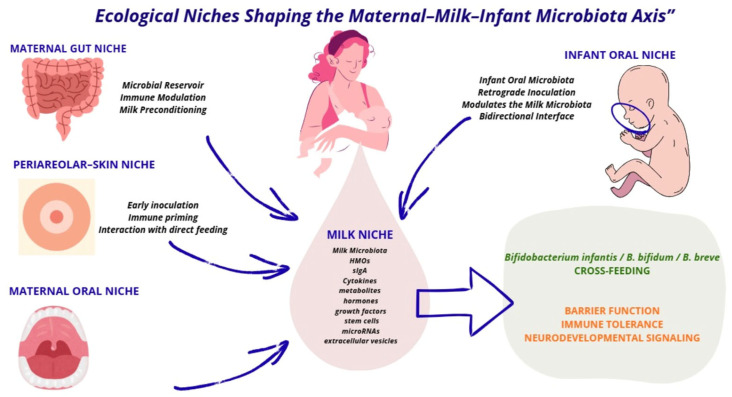
The materna–milk–infant microbiota axis. The figure illustrates breastfeeding as a dynamic ecological interface within the maternal–milk–infant microbiota axis. Human milk acts as a central biological hub integrating microbial communities from maternal gut, oral, skin, and mammary niches with infant oral and intestinal ecosystems. Microbial transfer occurs through enteromammary trafficking, direct breastfeeding, and retrograde inoculation during suckling. Human milk delivers microbes together with bioactive components that collectively shape early gut colonization, immune maturation, barrier development, and neurodevelopment during a critical window of plasticity.

Human milk is a uniquely adaptive biological fluid whose composition dynamically reflects maternal physiology, environmental context, and infant needs across lactation. Beyond its nutritional value, it is increasingly recognized as a living biological ecosystem. Human milk contains a distinct microbiota, human milk oligosaccharides (HMOs), metabolites, hormones, growth factors, antibodies, immune cells, cytokines, and extracellular vesicles that act in concert to guide early gut colonization, immune education, and neurodevelopment [4,5,6,7]. This integrated signaling system supports the establishment of a bifidobacteria-enriched gut microbiota, which has been consistently associated with enhanced barrier function, immune tolerance, reduced infection risk, and favorable neurodevelopmental trajectories [5,8] (Figure 1).

In this context, breastfeeding is increasingly understood not only as a preventive strategy, but also as a biologically active modulatory window capable of mitigating disrupted early-life microbial colonization. Conditions such as cesarean delivery, prematurity, neonatal intensive care admission, and early antibiotic exposure are associated with altered microbiota assembly and immune maturation, potentially increasing vulnerability to inflammatory and neurodevelopmental outcomes later in life [2,5,9,10]. Breastfeeding may partially buffer these perturbations by supporting microbial resilience and immune tolerance through multiple interconnected mechanisms.

Microbial transfer during breastfeeding occurs via the selective prebiotic effects of HMOs, enteromammary trafficking, retrograde inoculation during direct feeding, and immune-mediated signaling at the maternal–infant interface [11] (Figure 1). Importantly, these processes are not biologically fixed. Maternal diet, stress, environmental exposures, physical activity, and medical interventions can modulate milk composition and microbial signals, highlighting the ecological plasticity of breastfeeding biology [6,10].

Despite this complexity, public health discourse has traditionally emphasized breastfeeding duration and exclusivity as primary indicators of success. While valuable at a population level, this approach risks oversimplifying lactation biology and may inadvertently contribute to maternal guilt or overmedicalization [1,9]. A conceptual shift is therefore needed, moving from performance-based metrics toward a biologically informed understanding of breastfeeding as a dynamic, context-dependent system embedded within a broader ecological network.

Within this perspective, the One Health framework provides a valuable interpretative lens, allowing the identification of modifiable biological and environmental factors that may support microbial resilience and infant health while acknowledging individual variability and social constraints. The One Health concept recognizes the interconnectedness between human health, environmental systems, and microbial ecosystems, emphasizing that health outcomes emerge from interactions across these domains [12]. It is within this context that a microbiota-oriented decalogue for breastfeeding emerges as a tool to support conscious, evidence-informed practices aimed at optimizing early-life microbial and immune development without fostering maternal blame. Despite the growing body of research on human milk composition, early-life microbiota, and infant health, current evidence remains fragmented across disciplines, and integrative frameworks linking breastfeeding biology with ecological and environmental determinants are still limited [13,14]. In particular, the application of a One Health perspective to the maternal–milk–infant microbiota axis has not been systematically conceptualized. Therefore, this review aims to propose a microbiota-oriented breastfeeding decalogue grounded in a One Health framework, synthesizing current evidence to identify key biological and environmental domains influencing early-life microbial resilience and developmental trajectories.

## 2. Materials and Methods

### 2.1. Study Design

This study was conducted as a narrative review with a structured literature search, aimed at developing a microbiota-oriented Ten-Domain framework for breastfeeding within a One Health perspective. The objective was to integrate current evidence on the maternal–milk–infant microbiota axis and its potential implications for infant gastrointestinal, immune, and neurodevelopmental outcomes.

### 2.2. Literature Search

The structured literature search focused on studies published between January 2015 and January 2026 to capture the most recent evidence relevant to the maternal–milk–infant microbiota axis. Earlier landmark studies were also considered when necessary to provide essential biological background, historical context, or conceptual support for specific sections of the review; these references were identified through backward citation tracking and author-based hand-searching and were used to contextualize, rather than drive, the development of the final framework.

### 2.3. Study Selection

Approximately 200 records were initially identified through database searches. After removal of duplicates and title and abstract screening, 127 articles were assessed in full text for eligibility. Studies were included if they addressed biological, microbiological, immunological, environmental, or clinical aspects of the maternal–milk–infant microbial axis during breastfeeding.

### 2.4. Data Extraction

For each eligible study, key information was extracted, including study design, study population (maternal and/or infant), breastfeeding-related variables, microbiota outcomes, environmental or clinical determinants, and microbiota-modulating interventions such as probiotics. Data extraction and evaluation were conducted through iterative review of the literature by the authors.

### 2.5. Development of the Ten-Domain Framework

The Ten-Domain (Decalogue) framework was developed through an iterative conceptual synthesis of the selected literature. Determinants influencing maternal–infant microbial transmission and microbiota development during breastfeeding were identified and grouped into interconnected domains reflecting maternal factors, environmental and clinical exposures, and microbial modulation processes. These domains were progressively refined through author discussions to generate a ten-point framework representing the multidimensional interactions shaping early-life microbial ecosystems within a One Health perspective (Table 1).

Table 1 presents the studies cited in the manuscript that illustrate the evidence base supporting each domain of the One Health breastfeeding decalogue, including both the representative key studies and additional supporting studies referenced in the manuscript. Studies were selected based on relevance to the conceptual domain, scientific impact, and contribution to understanding the maternal–milk–infant microbiota axis. For each domain, multiple studies are included to reflect converging lines of evidence, highlighting both primary studies and additional literature that collectively support the principles of the decalogue.

## 3. Conceptual Framework: The Decalogue as a Microbiota-Oriented Framework

Given the biological complexity of breastfeeding and the multifactorial determinants shaping the maternal–milk–infant microbiota axis, there is a need for integrative frameworks capable of organizing heterogeneous mechanistic and clinical evidence without reducing it to prescriptive recommendations [1,2,6]. The decalogue proposed in this review is conceived as a microbiota-oriented conceptual framework rather than a set of clinical guidelines. Each of the ten points identifies a biologically relevant domain influencing microbial transfer, immune education, and metabolic and neurodevelopmental programming during breastfeeding. The points are not hierarchical and are intended to reflect interacting components of a complex adaptive system [5,10]. Within a One Health perspective, breastfeeding is approached as a modifiable biological process embedded in a broader ecological network. Methodologically, each point is examined through biological rationale, microbiota-related mechanisms, available clinical evidence, and contextual limitations, supporting informed clinical reasoning while avoiding maternal blame or overmedicalization [1,9,11].

## 4. The Ten Points of the Decalogue

The following ten points outline a microbiota-oriented conceptual framework for breastfeeding, summarizing key biological and environmental domains influencing early-life microbial development (Table 2).

### 4.1. Breast Milk as a Living Microbial and Signaling Ecosystem

Human milk represents the biological reference standard for early-life nutrition and is recommended as the exclusive source of nourishment for the first six months of life. Beyond its nutritional value, it is a dynamic and complex biological system that actively contributes to infant gastrointestinal, immune, and neurodevelopmental programming. Human milk functions as a living ecosystem in which microbial communities, immune factors, and bioactive molecules interact within a matrix that reflects maternal physiology, environment, and infant needs [15]. A growing body of evidence links exclusive and prolonged breastfeeding to a reduced incidence of allergic diseases and respiratory infections. In the CHILD Cohort Study, distinct patterns of milk composition and microbial colonization mediated the protective effect of breastfeeding on asthma risk, whereas early discontinuation was associated with premature microbiota maturation and enrichment of taxa such as *Ruminococcus gnavus*, previously linked to immune dysregulation [36]. These findings highlight the role of sustained breastfeeding in supporting a regulated microbial succession.

Human milk composition evolves from colostrum to mature milk, reflecting the changing physiological needs of the infant. Colostrum is particularly enriched in immune and bioactive factors and shows higher protein content, especially in mothers of preterm infants [16]. Despite common developmental patterns, milk composition displays marked interindividual variability influenced by infant characteristics (gestational age, mode of delivery, antibiotic exposure) and maternal factors, including diet, body mass index, lifestyle, psychological status, and environmental exposures [17,18].

Human milk also constitutes a major source of microbial exposure, with exclusively breastfed infants ingesting approximately 10^5^–10^7^ microbial cells daily [3]. The milk microbiota has been proposed to originate from maternal gut, skin, oral, and mammary niches through enteromammary trafficking, retrograde flow during suckling, and oromammary pathways, commonly including *Streptococcus*, *Staphylococcus*, *Bifidobacterium*, *Veillonella*, *Rothia*, and Bacteroides. Antibiotic exposure and maternal obesity are associated with reduced microbial diversity and depletion of beneficial bifidobacteria. Feeding modality further influences microbial transfer, with direct breastfeeding favoring oral-associated taxa compared with expressed milk [19]. Beyond its microbial content, human milk delivers immune and signaling molecules such as lactoferrin, lysozyme, antimicrobial peptides, secretory IgA and IgG, cytokines including TGF-β, complement components, microRNAs, and milk fat globule membranes, all contributing to immune tolerance, epithelial integrity, and neurodevelopment [20,21,22,23].

Human milk oligosaccharides (HMOs) represent a central functional component, acting as selective substrates for infant-type bifidobacteria and promoting short-chain fatty acid production, barrier maturation, and immune regulation [24,25,26]. Through microbiota-mediated pathways, HMOs and associated microbial metabolites influence neurodevelopmental processes, including sialic acid availability, GABA production, and tryptophan–kynurenine metabolism, with implications for cognitive and behavioral outcomes [27,28,29,30,31,32].

Collectively, these data support the interpretation of human milk as a living, plastic, and biologically active ecosystem that shapes early-life microbial assembly and developmental trajectories within the maternal–milk–infant axis.

### 4.2. Direct Breastfeeding and Microbial Transfer

Direct breastfeeding represents a biologically relevant route for microbial transfer from mother to infant, supporting early gut colonization through mechanisms that extend beyond milk composition alone. Compared with expressed or pumped milk, direct feeding minimizes oxygen exposure and environmental contamination, favoring the transfer of viable maternal microbes during a critical window of microbial assembly [4,11]. Several complementary mechanisms contribute to this process. In addition to the delivery of milk-associated bacteria, direct breastfeeding enables retrograde inoculation, whereby infant oral microbes interact with the mammary microbiota, potentially shaping milk microbial composition through bidirectional exchange [11,34]. This dynamic interaction occurs within a broader signaling context involving immune cells, antibodies, and bioactive molecules that may influence microbial survival and immune education at the maternal–infant interface [7]. From a microbiota perspective, direct breastfeeding has been associated with a higher likelihood of establishing a bifidobacteria-dominated gut ecosystem, a feature consistently linked to improved barrier function, immune tolerance, and reduced infection risk in early life [5,8,35]. Observational studies suggest that infants fed directly at the breast may display greater microbial richness and more coherent immune signaling patterns compared with those receiving predominantly expressed milk; however, these associations derive from observational data, and causal relationships remain difficult to establish [6,7,33]. Importantly, direct breastfeeding is not always feasible due to medical, social, or logistical constraints. Available evidence indicates that expressed breast milk retains substantial biological value, including HMOs, immune factors, and microbial components, even if some aspects of microbial transfer may be attenuated [7]. Within a One Health framework, direct breastfeeding should therefore be understood as a biologically meaningful opportunity rather than a rigid standard, acknowledging variability while preserving a non-prescriptive, context-sensitive approach.

### 4.3. Feeding Rhythms, Responsiveness, and Circadian Biology

Early life represents a sensitive window for circadian system maturation, during which feeding rhythms and mother–infant interactions contribute to the entrainment of emerging biological clocks. In humans, the sleep–wake circadian rhythm typically consolidates within the first three months of life; however, its maturation shows considerable interindividual variability and may be influenced by feeding practices. A retrospective study comparing exclusive breastfeeding with mixed feeding showed that exclusively breastfed infants exhibited a more clearly defined 24 h rest–activity rhythm by six weeks of age, paralleled by stronger maternal circadian rhythmicity, whereas rhythmicity in mixed-fed dyads emerged later [37].

One biological basis for this association lies in the circadian variation in human milk composition. Systematic analyses have demonstrated diurnal fluctuations in multiple milk components, including hormones, metabolites, and nucleotides [38,39,120]. The non-protein nitrogen fraction of human milk, particularly nucleotides involved in sleep homeostasis, exhibits marked circadian variation. Sánchez et al. reported higher nocturnal concentrations of adenosine 5′-monophosphate (5′AMP), guanosine 5′-monophosphate (5′GMP), and uridine 5′-monophosphate (5′UMP), while cytidine and inosine nucleotides peaked during daytime hours [40]. These nocturnal increases may contribute to the sleep-promoting effects of nighttime breast milk, particularly in infants whose endogenous melatonin secretion remains immature until 3–5 months of age.

Melatonin represents a central chronobiological signal transferred through breastfeeding. While classically produced by the pineal gland, melatonin is also synthesized in extra-pineal sites, including the gut, immune cells, and placenta, with postprandial intestinal production reaching concentrations far exceeding nocturnal pineal peaks [41]. Melatonin biosynthesis from tryptophan proceeds via serotonin and N-acetylserotonin (NAS), both of which exert antioxidant and neurotrophic effects, with NAS mimicking brain-derived neurotrophic factor signaling through TrkB activation [42]. Maternal nocturnal melatonin surges are reflected in breast milk, facilitating hormonal transfer to the infant.

Experimental data further suggest that melatonin modulates gut microbial composition and intestinal maturation. In perinatal animal models, melatonin exposure was associated with reduced oxidative stress, autophagy, and inflammation, alongside increased abundance of short-chain fatty acid–producing genera such as *Allobaculum*, *Bifidobacterium*, and *Faecalibaculum* [43]. Collectively, these findings support the concept that feeding rhythms and responsive breastfeeding convey temporal biological signals that may influence circadian entrainment, intestinal barrier function, and microbiota development during early life.

### 4.4. Maternal Diet and Microbial Biodiversity

Among the numerous maternal and infant-related factors associated with the composition of the human milk microbiome, maternal diet has emerged as a potentially modifiable determinant. However, current evidence on the influence of maternal diet on the human milk microbiome remains limited and highly heterogeneous. Differences in dietary assessment methods, milk sampling protocols, and microbiome analysis techniques substantially hinder comparability across studies, highlighting the need for a cautious and critical appraisal of the available literature [44].

Within a One Health perspective, maternal diet should be interpreted as one component of a broader ecological system interacting with maternal physiology, perinatal exposures, feeding practices, and environmental context. Consistent with evidence from other domains of the maternal–milk–infant axis, dietary effects appear modest when compared with stronger determinants such as mode of delivery, antibiotic exposure, maternal body mass index, and feeding modality [1,45].

In this context, Taylor et al. [45] conducted a scoping review of 19 observational and interventional studies examining associations between maternal diet and the human milk microbiome and/or the infant gut microbiome. Ten studies focused on the human milk microbiome, eleven on the infant gut microbiome, and only two assessed both compartments simultaneously. Overall, maternal intake of dietary fat, fiber, micronutrients, plant-based foods, and ultra-processed foods was variably associated with specific microbial taxa. However, dietary effects were generally modest and less influential than other determinants, including mode of delivery, antibiotic exposure, and maternal body mass index, and evidence supporting a clear mediation of infant gut microbiota changes through human milk remained limited.

Further insight is provided by Cortes-Macías E et al. [46], who demonstrated that maternal diet influences the composition and diversity of the human milk microbiota in interaction with perinatal factors. Firmicutes, Proteobacteria, and Actinobacteria were the predominant phyla, with *Streptococcus* and *Staphylococcus* as the most abundant genera. A dietary pattern characterized by higher intake of fiber, plant protein, carbohydrates, and polyphenols was associated with greater microbial diversity and increased abundance of *Bifidobacterium*, whereas higher consumption of animal protein and lipids was associated with reduced abundance of taxa considered beneficial and increased *Gemella*. These associations were further modulated by cesarean delivery and antibiotic exposure.

Overall, available evidence suggests that maternal diet may contribute to shaping microbial biodiversity and ecological resilience of human milk rather than exerting deterministic effects on specific taxa. Within a microbiota-oriented One Health framework, dietary diversity and quality during lactation should therefore be viewed as supportive ecological factors embedded within a complex, context-dependent maternal–milk–infant system [2,10].

### 4.5. Oral and Skin Microbiota as Sources of Colonization

Recent research has reshaped our understanding of neonatal microbial acquisition, highlighting maternal oral and periareolar microbiota as critical sources for seeding the infant oral and gut ecosystems [47]. The periareolar region represents a unique microbial niche, integrating bacteria from maternal skin, the oral cavity, mammary-associated communities, and the infant’s mouth through direct contact and bidirectional exchange during breastfeeding [48,49].

Maternal oral microbes may reach the breast through multiple routes, including oral–mammary translocation, the entero-mammary pathway, and direct saliva contact [49]. During feeding, infant oral colonization occurs through close contact with periareolar skin and saliva exchange [50], while breast milk delivers oral-origin bacteria to the infant gut [51]. This bidirectional microbial flow, known as retrograde inoculation, supports a dynamic microbial dialogue at the maternal–infant interface, with skin-associated microbes contributing to early immune priming [52].

Importantly, the infant oral cavity represents not only a primary site of colonization but also a key reservoir for the developing respiratory microbiota. Early-life lung microbial communities are largely derived from the oropharynx through microaspiration, making maternal oral microbiota an indirect but biologically relevant determinant of respiratory microbial assembly and immune programming [53,54].

Strain-level metagenomic analyses confirm vertical transmission, revealing identical oral and skin bacterial strains shared between mothers and infants [55], with substantial oral [56,57] and skin [58] microbiome overlap. The efficiency of this transfer is modulated by maternal oral hygiene [59], nipple microbiota [60], mastitis [61], antibiotics [62], and environmental, dietary, and lifestyle factors [63,64]. Within a One Health framework, maternal oral health thus emerges as a key, yet often overlooked, determinant of intergenerational microbial and immune continuity.

### 4.6. Breast Care and Mammary Microbial Homeostasis

Human milk is a dynamic biological fluid providing nutrition, bioactive molecules, immunological factors, and a resident microbiota critical for neonatal gut colonization, metabolism, immune-neuroendocrine development, and mammary health [65]. Mammary eubiosis supports lactational health, whereas disruption favors opportunistic pathogens such as *Staphylococcus aureus* [65]. Maternal diet, particularly macronutrient composition and energy intake, may modulate human milk oligosaccharides (HMOs), including fucosylated species, with consequences for microbial metabolic capacity [66].

The human milk microbiota (HMM) derives from multiple sources: the entero-mammary pathway, maternal skin, and bidirectional exchange with the infant oral microbiome, serving as an early microbial inoculum for the neonatal gut [65]. The areola-nipple skin is a key ecological interface; breast care practices that alter barrier integrity or local microclimate may influence microbial balance. During sub-acute mastitis, bacterial richness and diversity decrease and recover after symptom resolution, consistent with a polymicrobial dysbiotic process [67,68]. Maintaining balance among dominant genera (*Staphylococcus*, *Streptococcus*, *Corynebacterium*) is essential for mammary homeostasis [68,69].

Environmental factors such as excessive cleansing, harsh detergents, or occlusive clothing can modify humidity and barrier function, affecting cutaneous microbial communities and susceptibility to inflammation [65]. Intrapartum antibiotic prophylaxis reduces microbial diversity and alters composition, highlighting the need for longitudinal multi-omic studies [68,70]. Probiotics have been investigated as potential preventive and adjunctive strategies [71]. Selected strains such as *Lactobacillus salivarius* PS2 and *L. fermentum* CECT5716 have shown reductions in mastitis incidence, staphylococcal load, and inflammatory markers in clinical studies [72,73]. Other candidates, including oral strains such as *Streptococcus salivarius*, may support mammary and oral eubiosis and inhibit pathogenic biofilms, although direct evidence in lactational mastitis remains limited [74,75]. However, findings are heterogeneous, and larger well-designed randomized trials are required to define optimal strains, timing, and dosing before routine clinical recommendations can be made.

### 4.7. Environmental Exposure, Nature, and Physical Activity

The exposome, encompassing cumulative environmental exposures across the lifespan, significantly influences breast milk composition, human milk microbiota (HMM), and infant health within One Health and Planetary Health frameworks [72]. Maternal exposures during pregnancy and lactation shape offspring immune development through antibody transfer, microbiome modulation, and immune mediators conveyed via breastfeeding [76], while environmental risks such as pollution and chemical contaminants contribute to long-term health vulnerability [77].

Pollutants bioaccumulate in breast milk in a geography- and diet-dependent manner. Black carbon nanoparticles, markers of traffic-related exposure, have been detected in human milk and correlate with residential particulate exposure [78]. Persistent organic pollutants, microplastics, and heavy metals also accumulate over time, although direct links to infant gut dysbiosis remain incompletely defined [79,80,81,82,83,84]. Air pollution exposure has been associated with reduced HMO diversity and altered infant gut microbiota profiles, whereas residence in greener environments correlates with increased HMO diversity, suggesting beneficial environmental microbial exposures [85,86,87]. Maternal physical activity may further modulate milk bioactivity, including adiponectin levels implicated in infant metabolic programming, although evidence remains limited [88,89,90]. Disentangling interactions between environmental exposures, maternal physiology, HMM dynamics, and infant outcomes remains challenging, with most evidence derived from cross-sectional studies. Longitudinal exposomic approaches are therefore needed to clarify causal pathways [34,91,92,93,94,95]. Within a One Health perspective, the exposome should not be conceptualized solely as a source of toxic burden, but as a dynamic ecological interface shaping the maternal–milk–infant microbiota axis across generations.

### 4.8. Stress, Neuroendocrine Signaling, and Microbiota

Maternal stress during pregnancy and postpartum activates the hypothalamic–pituitary–adrenal (HPA) axis and sympathetic pathways, altering hormonal, immune, and metabolic signaling that can influence both human milk (HM) composition and microbial ecology [49,97]. Fluctuations in cortisol, catecholamines, and oxytocin may affect mammary gland physiology, immune components, and microbial transfer through the proposed entero-mammary pathway.

Maternal stress during pregnancy and postpartum is associated with adverse neurodevelopmental and health outcomes in offspring [98]. One hypothesized mechanism involves stress-related changes in HM microbiota composition. Higher maternal psychosocial stress in full-term pregnancies has been associated with significantly lower bacterial diversity in HM at three months postpartum [99]. In a cohort of 51 healthy mothers, microbial diversity increased from week 2 to week 12 in women with low psychosocial distress, characterized by a decrease in *Staphylococcus* and increases in *Lactobacillus*, *Acinetobacter*, and *Flavobacterium*, whereas diversity remained relatively stable in highly distressed mothers [99]. Importantly, prenatal maternal stress has also been linked to alterations in early infant gut microbial colonization. In a prospective mother-infant cohort study, Zijlmans et al. [96] reported that higher maternal prenatal stress and cortisol levels were associated with distinct infant gut microbiota profiles during early life, including reduced abundance of beneficial taxa such as *Bifidobacterium* and increased representation of potentially pro-inflammatory bacteria.

Similarly, a prospective observational study comparing 23 highly stressed lactating women with 69 controls reported differences in HM β-diversity, with decreased abundance of *Streptococcus*, *Gemella*, and *Veillonella*, and increased *Staphylococcus*, *Corynebacterium*, and *Acinetobacter* in the stressed group [100]. These findings may partly reflect stress-induced alterations in the maternal gut microbiota transmitted via the entero-mammary pathway [49,99]. In turn, early HM microbial changes could influence initial gut colonization of breastfed newborns, a critical window for immune and metabolic programming. However, evidence remains largely associative and confounded by maternal, environmental, and behavioral factors, and few studies simultaneously assess HM, maternal gut, and infant microbiota. A recent study evaluating all three compartments did not find a significant impact of stress-related HM microbiota changes on maternal or infant gut microbiota trajectories [101].

Overall, maternal stress likely exerts multidimensional effects on lactational biology, encompassing neuroendocrine signaling, immune factors, and microbial composition, yet the relative contribution of each pathway to infant outcomes remains uncertain. Large longitudinal studies integrating neuroendocrine, nutritional, microbial, and developmental data are needed to clarify causal relationships within this complex mother-infant system.

### 4.9. Protecting the Microbiota During Medical Interventions

Early life and perinatal exposure to medications can disrupt the normal maturation pattern of the gut microbiota [103]. Among these, β-lactam antibiotics such as amoxicillin, amoxicillin-clavulanic acid, and ampicillin are among the most frequently prescribed during pregnancy, intrapartum care, breastfeeding, and in infancy, with the potential to perturb microbial colonization patterns [102,110]. Antibiotic exposure reduces intestinal colonization of Bifidobacteria, alters key taxa such as Bacteroides, and increases Proteobacteria, promoting a proinflammatory colonic milieu associated with increased risks of atopy, obesity, inflammatory bowel disease, type 1 diabetes, and reduced vaccine responses [102,104,105,106]. Other drugs, such as acid suppressants (PPIs/H2 antagonists), can also modify gut microbiota by increasing oral-to-gut microbial transmission [107] and have been linked to necrotizing enterocolitis, sepsis, and metabolic sequelae in infants [108].

While antibiotic therapy often cannot be avoided, a conscious and targeted approach under medical supervision is recommended to minimize unnecessary exposure and protect microbiota integrity. Probiotic adjuvant strategies can help mitigate antibiotic-induced dysbiosis. Certain probiotics demonstrate resistance profiles or intrinsic antibiotic insensitivity suitable for co-administration during β-lactam therapy. A strain of *Bifidobacterium breve* PRL2020 shows resistance to amoxicillin, amoxicillin-clavulanate, and ampicillin under EFSA criteria, reducing antibiotic-induced dysbiosis without horizontal gene transfer [109]. Yeast probiotics are intrinsically insensitive to antibiotics; for example, *Saccharomyces boulardii* CNCM I 745 effectively reduces antibiotic-associated diarrhea and supports microbiota resilience [111]. Spore-forming bacteria such as *Bacillus clausii* and *Clostridium butyricum* exhibit reduced sensitivity to antibiotics during sporulation, allowing partial preservation of gut colonization and recovery upon cessation of therapy [112,113].

Probiotic administration may be considered in three scenarios: (i) to the mother during antibiotic therapy in pregnancy or lactation, supported by evidence that maternal probiotic supplementation modulates breastmilk and infant gut microbiota [114,115]; (ii) directly to the infant during antibiotic exposure, with clinical data showing that concomitant probiotic use during antibiotics can favourably influence microbial abundance in neonates [116]; (iii) to a breastfed infant of a mother receiving antibiotics, aiming to buffer indirect dysbiosis via milk microbial transfer, a principle consistent with observed clinical probiotic use to prevent antibiotic-associated effects [117].

Clinical application should consider strain specificity, timing, dosage, safety, and antibiotic resistance profiles when selecting probiotic adjuncts to preserve microbiota resilience during medically necessary interventions.

### 4.10. Complementary Feeding with Continued Breastfeeding

The complementary feeding period, spanning approximately from 6 to 24 months of age, represents a critical developmental window in which infants transition from an exclusively milk-based diet to a progressively more complex dietary pattern required to meet increasing nutritional needs [119]. According to the World Health Organization, complementary foods should be introduced when breast milk alone no longer meets energy and nutrient requirements, while continued breastfeeding is recommended alongside complementary feeding up to two years of age or beyond [118]. This transitional phase coincides with sensitive windows of gut microbiota and immune system maturation [119]. Longitudinal studies have demonstrated that gut microbiota development follows sequential stages, including a developmental phase, a transitional phase, and a stabilization phase during early childhood. Breast milk acts as the primary ecological driver in early life, promoting a bifidobacteria-dominated microbiota, whereas cessation of breastfeeding is associated with accelerated maturation toward an adult-like microbial configuration enriched in Firmicutes [121].

Within this framework, the gradual introduction of complementary foods during continued breastfeeding represents a biologically coherent strategy to support microbial ecological succession. Breast milk continues to provide bioactive compounds, immune factors, and selective substrates, while complementary foods introduce novel fermentable components that promote microbial diversification. Consistent with this model, the introduction of solid foods has been associated with increased alpha diversity and progressive restructuring of gut microbial composition [119]. This transition typically involves a relative reduction in Bifidobacteriaceae and an increase in taxa such as Lachnospiraceae, Ruminococcaceae, and *Clostridium* spp., although associations with specific food groups remain inconsistent.

Importantly, dietary diversity during complementary feeding appears to correlate with increased microbial richness; however, conclusions are limited by heterogeneity in dietary assessment, lack of controlled feeding studies, and variability in microbiota profiling methods [119]. From a One Health perspective, continued breastfeeding during complementary feeding may act as a buffering ecological factor, supporting a gradual and adaptive microbiota transition while linking maternal nutrition, infant microbial development, and long-term immune–metabolic resilience [118,121].

### 4.11. Interconnectedness of the Decalogue Domains

Although presented as distinct conceptual domains, the ten points of the microbiota-oriented breastfeeding decalogue should be interpreted as interconnected elements of a broader ecological system. Early-life microbial assembly emerges from the dynamic interaction between maternal physiology, infant feeding practices, environmental exposures, and psychosocial factors rather than from isolated determinants. Evidence from microbiome research increasingly supports this integrative perspective, showing that nutritional, biological, and environmental influences collectively shape microbial and immune development during the first years of life [13,14]. Within this framework, the decalogue is intended not as a set of independent recommendations but as a conceptual model aligned with the One Health perspective, which emphasizes the interdependence of human, environmental, and microbial health systems [12].

## 5. Probiotics During Breastfeeding: Opportunities and Pitfalls

The use of probiotics during breastfeeding has gained increasing attention as a strategy to support infant gut colonization and immune maturation during a critical window of microbial plasticity. Their application appears biologically justified in conditions associated with interference with early microbial assembly, including cesarean delivery, prematurity, neonatal intensive care exposure, and antibiotic use during pregnancy or lactation [2,10]. Beyond perinatal and neonatal perturbations, probiotic interventions may also be relevant in the presence of maternal dysbiosis. Women affected by chronic immunological or inflammatory conditions frequently display altered gut microbial profiles, often characterized by reduced abundance of bifidobacteria and altered immune-microbial signaling, with potential consequences for vertical microbial transmission [122,123].

### 5.1. Maternal Supplementation: Timing and Target

A critical and often under-discussed variable is the timing of maternal supplementation. When probiotics are administered to the mother to modulate the maternal–milk–infant axis, initiation during the last trimester of pregnancy appears biologically strategic. Several randomized controlled trials suggest that probiotic supplementation started in late pregnancy and continued through lactation may modulate maternal gut microbiota, influence breast milk immune composition, and shape infant gut colonization patterns [124,125,126]. These findings support the concept that effective microbial transfer during breastfeeding requires adequate maternal colonization before delivery. From a mechanistic perspective, prenatal probiotic supplementation has been associated with modulation of maternal immune signaling, including altered cytokine profiles and increased secretory IgA levels in breast milk, suggesting an indirect but biologically meaningful pathway linking maternal microbiota, milk composition, and infant immune programming [125,127,128]. This reinforces the view of breastfeeding as a continuum beginning before birth, rather than an isolated postnatal event. A further practical consideration concerns the target of supplementation. Probiotics may be administered to the mother, the infant, or both. Maternal supplementation acts upstream, potentially influencing milk microbiota composition, immune signaling, and microbial transfer [6,7]. Infant supplementation may be appropriate when dysbiosis is already established, breastfeeding is partial or delayed, or maternal supplementation is not feasible. Current evidence does not support a universally superior strategy, underscoring the need for individualized, context-dependent decision-making.

### 5.2. Strain Specificity and Ecological Engraftment

Strain specificity represents a central biological constraint. Infant-type bifidobacteria differ markedly in metabolic capacity and ecological behavior. *Bifidobacterium longum* subsp. *infantis* is highly specialized for intracellular utilization of human milk oligosaccharides (HMOs) and shows optimal colonization in exclusively breastfed infants with adequate HMO exposure [5,35,129]. In contrast, *Bifidobacterium bifidum* expresses extracellular glycosidases and mucinolytic enzymes, enabling utilization of both HMOs and intestinal mucin and supporting cross-feeding with other bifidobacteria, including *B. breve* [34,130]. Its sialidase activity may additionally increase sialic acid availability, a molecule implicated in early neurodevelopment [131]. Lactobacilli, such as *Lactobacillus reuteri* and *Lacticaseibacillus rhamnosus*, exert complementary functions by supporting mucosal barrier integrity, immune modulation, and pathogen exclusion, contributing to intestinal resilience during early life without reconstructing an infant-type ecosystem [7,9], an important ecological consideration is that reported clinical effects of *Lactobacillus*/*Lacticaseibacillus* supplementation during early life do not necessarily correspond to stable intestinal engraftment, particularly in the context of human milk feeding. In preterm infants exposed to routine NICU probiotic regimens combining *Bifidobacterium* and *Lactobacillus* species, longitudinal strain tracking has demonstrated a clear asymmetry in persistence: *Bifidobacterium* strains are more consistently detectable and may remain measurable weeks after supplementation, whereas *Lactobacillus* signals often decline rapidly following discontinuation and are inconsistently detected over time, suggesting limited or non-durable engraftment [132,133]. In term infants, supplementation with *Lactobacillus reuteri* DSM 17938 has likewise been associated with variable and frequently low detection rates at the end of intervention, despite reported clinical or inflammatory changes [134]. From a mechanistic standpoint, this pattern is biologically plausible. Compared with infant-adapted bifidobacteria—such as *Bifidobacterium longum* subsp. *infantis* and *B. bifidum*—many lactobacilli possess a comparatively restricted repertoire of glycosidases and transport systems required for efficient utilization of the structurally diverse human milk oligosaccharide (HMO) pool, which represents a dominant ecological driver in the breastfed infant gut. In an HMO-rich environment, bifidobacteria specialized in HMO metabolism may therefore achieve a competitive advantage, expand through cross-feeding networks, and establish stable ecological niches, whereas lactobacilli may remain numerically minor, intermittently detectable, and less likely to persist. Accordingly, when Lactobacillus/Lacticaseibacillus supplementation during breastfeeding is associated with clinical benefit, these effects may plausibly reflect transient functional mechanisms—such as metabolite production, modulation of mucosal immune signaling, enhancement of epithelial barrier integrity, or competitive interactions during gastrointestinal transit—rather than durable reconstruction of an infant-type microbial ecosystem. Distinguishing between functional impact and ecological engraftment is therefore essential when interpreting probiotic trials in early life, as probiotic efficacy and stable colonization represent related but non-equivalent biological outcomes.

### 5.3. Early-Life Microbiota and Vaccine Responses

Another clinically relevant implication of early-life microbiota modulation during breastfeeding concerns vaccine responsiveness. Accumulating evidence indicates that early antibiotic exposure, which perturbs microbial assembly and in infants reduces bifidobacterial abundance, is associated with impaired humoral responses both in experimental testing and in routine pediatric vaccinations [135,136]. In a longitudinal cohort study, early *Bifidobacterium* abundance at 6–15 weeks of life was positively associated with enhanced systemic and mucosal responses to multiple vaccines, including BCG, tetanus toxoid, hepatitis B, and oral polio virus, with differences in predicted antibody titers and T-cell responses between low (10th percentile) and high (90th percentile) bifidobacterial abundance ranging from 42% to over 100% depending on the antigen [106]. Notably, colonization with *Bifidobacterium* at the time of vaccination was associated with more sustained vaccine-specific memory T-cell and antibody responses at 2 years of age, supporting a durable immunologic imprinting effect. Mechanistically, this association is biologically plausible. Infant-type bifidobacteria are dominant producers of acetate in the breastfed gut, and short-chain fatty acids (SCFAs) have been shown to modulate immune cell metabolism and differentiation. Acetate can enhance mitochondrial oxidative phosphorylation and ATP generation, processes that are energetically essential for plasmablast differentiation and high-rate immunoglobulin synthesis [137]. In this context, a bifidobacteria-enriched ecosystem may provide metabolic substrates that support plasma cell bioenergetics and antigen-specific antibody production, thereby functionally linking early microbial ecology with vaccine-induced adaptive immunity. These observations have led some authors to propose that microbiota-targeted strategies—particularly bifidobacterial supplementation—around the time of vaccination could represent a rational approach to enhance vaccine immunogenicity in early life, although controlled interventional trials are still required before routine implementation can be recommended [138].

Overall, probiotics during breastfeeding should be regarded as ecologically informed, timing-sensitive tools, not as universal solutions. Their use must consider maternal microbial status, prenatal and postnatal timing, breastfeeding modality, and in-fant clinical context, avoiding reductionist or supplement-centered approaches that oversimplify early-life microbial programming.

## 6. Ethical and Clinical Implications

Breastfeeding occupies a complex ethical space at the intersection of biology, public health, social norms, and individual experience. Although its benefits for infant gastrointestinal, immune, and neurodevelopmental health are well documented, the translation of this evidence into clinical recommendations requires caution to avoid oversimplification, maternal guilt, or overmedicalization [1,9,139], consistent with international public health frameworks promoting respectful and supportive breastfeeding practices (WHO, 2017) [140].

A microbiota-oriented One Health perspective supports a conceptual shift from performance-based metrics of breastfeeding success toward a context-sensitive biological framework. Within this view, breastfeeding is not framed as a moral obligation or a uniform standard, but as a dynamic biological system influenced by maternal health, environmental exposures, psychosocial stressors, and healthcare practices [2,10]. This approach promotes respect for maternal autonomy while preserving scientific rigor.

The use of probiotics during breastfeeding highlights key ethical and clinical tensions. While targeted probiotic interventions may be justified in specific contexts, such as disrupted microbial implantation, maternal dysbiosis, or increased infant vulnerability, their use should not be portrayed as universally necessary or as a prerequisite for adequate breastfeeding [6,7]. Reductionist, supplement-centered narratives risk transferring responsibility for biological or structural determinants of health onto individual mothers, contrary to One Health principles [1,9].

Current international clinical guidelines provide a cautious and context-dependent perspective regarding probiotic use in early life and during breastfeeding. Position statements from the European Society for Paediatric Gastroenterology, Hepatology and Nutrition (ESPGHAN) indicate that probiotic supplementation may be considered in specific clinical conditions, such as prevention of necrotizing enterocolitis in preterm infants or management of selected gastrointestinal disorders, but emphasize that recommendations are strain-specific and condition-specific rather than universal [141,142]. Importantly, these guidelines do not recommend routine probiotic supplementation for healthy breastfeeding mother-infant dyads.

Similarly, the World Gastroenterology Organisation Global Guidelines on Probiotics and Prebiotics highlight that probiotic efficacy depends on the specific microbial strain, dosage, and clinical context, and therefore should not be generalized as a universal adjunct to breastfeeding or early-life nutrition [143]. These recommendations reinforce the need for careful clinical evaluation before considering probiotic use in breastfeeding contexts. From a clinical standpoint, a microbiota-informed approach favors personalized counseling and shared decision-making, integrating maternal clinical history, mode of delivery, infant condition, and breastfeeding feasibility. Such an approach aligns with ethical principles of proportionality, non-maleficence, and contextualized care, and discourages rigid protocols in favor of adaptive clinical reasoning [10]. Several limitations should also be acknowledged. Evidence regarding probiotic use during breastfeeding remains heterogeneous, with substantial variability in probiotic strains, study designs, and clinical populations. Many studies focus on specific neonatal conditions, preterm infants, or formula-fed populations rather than exclusively breastfed infants, limiting generalizability. Furthermore, the long-term ecological effects of probiotic supplementation on the developing infant microbiome remain incompletely understood. As emphasized in recent consensus and guideline documents, probiotic effects are often strain-specific and context-dependent, and evidence across clinical settings remains uneven [143,144]. These uncertainties support a cautious and individualized clinical approach consistent with the One Health framework.

Ultimately, framing breastfeeding as a modifiable biological process rather than a normative benchmark allows clinicians and families to engage with early-life nutrition in a scientifically grounded, ethically responsible, and compassionate manner, consistent with the preventive and integrative goals of the One Health framework.

## 7. Future Directions and Research Gaps

Despite growing recognition of breastfeeding as a dynamic microbiota-mediated system, several research gaps remain. First, most available evidence derives from cross-sectional or observational studies, limiting causal inference. Longitudinal, multi-omic designs integrating maternal gut, milk, and infant microbiota with immune, metabolic, and neurodevelopmental outcomes are needed to clarify directionality and effect size within the maternal–milk–infant axis.

Second, methodological heterogeneity in milk sampling, microbial sequencing, dietary assessment, and stress measurement hampers comparability across studies. Standardized protocols and strain-level metagenomics should be prioritized to characterize vertical transmission and functional microbial pathways better.

Third, the interaction between environmental exposures, psychosocial stress, medical interventions, and milk ecology requires integrative exposomic approaches capable of disentangling cumulative and synergistic effects.

Fourth, probiotic strategies during lactation demand rigorously designed randomized controlled trials addressing strain specificity, timing, dosage, safety, and long-term infant outcomes, avoiding reductionist supplementation paradigms. Finally, future research should explore transgenerational implications and equity-related determinants within a One Health framework, ensuring that biologically informed recommendations remain context-sensitive and do not contribute to maternal overmedicalization or blame.

## 8. Conclusions

Breastfeeding represents a critical developmental window during which maternal physiology, microbial ecosystems, and environmental exposures converge to influence infant gastrointestinal, immune, and neurodevelopmental trajectories. Human milk functions as a dynamic biological system delivering microbes, microbial substrates, and immune mediators that participate in early-life programming. In this context, breastfeeding may help support microbial continuity when early perturbations, such as cesarean delivery, prematurity, neonatal intensive care exposure, antibiotic use, or maternal dysbiosis, affect vertical microbial transmission during a period of heightened developmental plasticity.

The microbiota-oriented One Health Ten-Domain (Decalogue) framework proposed in this review integrates maternal, environmental, and microbial determinants that shape early-life microbial assembly. Maternal factors (e.g., diet, metabolic status, and microbiota composition) interact with environmental and clinical influences such as delivery mode, healthcare practices, and microbial exposures, while microbial processes, including vertical transfer through breastfeeding and targeted modulation strategies such as probiotics, contribute to ecosystem resilience. By conceptualizing these domains as interconnected influences, the Decalogue provides a structured framework to support microbiota-aware breastfeeding practices and early-life preventive strategies.

## Figures and Tables

**Table 1:** Evidence Supporting the One Health Breastfeeding Decalogue.

Authors (Ref.)	Year	Journal	Population/Model	Study Design	Key Finding Relevant to Breastfeeding-Microbiota Axis	Decalogue Domain Supported	Additional Supporting Studies Cited
Fernández et al. [4]	2013	Pharm Res	Human milk microbiome	Review	Milk hosts microbial ecosystem shaping infant gut	**1. Breast milk** as a living microbial and signaling ecosystem	[10,14,15,16,17,18,19,20,21,22,23,24,25,26,27,28,29,30,31,32]
Moossavi et al. [6]	2019	Cell–Host Microbe	393 Mother-infant dyads	Prospective cohort study (within a birth cohort)	Milk microbiota varies with maternal factors		
Lyons et al. [7]	2020	Nutrients	Human milk microbiome	Review	Milk microbes and HMOs shape colonization		
Milani et al. [5]	2017	Microbiol Mol Biol Rev	Infant gut microbiota	Review	Early microbial colonization strongly influenced by breastfeeding-derived microbes		
Pannaraj et al. [33]	2017	JAMA Pediatr	25 Mother-infant dyads	Prospective longitudinal Cohort	Vertical microbial transfer milk → infant gut	2. **Direct breastfeeding** and microbial transfer	[4,5,6,7,8,11,33,34,35]
Shenhav et al. [36]	2024	Cell	2227 children from the CHILD Cohort Study	Prospective longitudinal cohort study	Breastfeeding duration shapes microbial succession in infancy		
Tamburini et al. [14]	2016	Nat Med	Early life microbiome	Review	Feeding ecology drives microbiota		
Victora et al. [1]	2016	Lancet	Global population	Systematic review	Exclusive Breastfeeding linked to immune benefits	3. **Feeding rhythms**, responsiveness, and circadian biology	[14,37,38,39,40,41,42,43]
Donald & Finlay [9]	2023	Nat Rev Immunol	Host–microbiome interaction	Review	Feeding patterns affect immune–microbiome interactions		
Fasano et al. [10]	2024	Gut Microbes	Maternal–infant microbiota	Review	Maternal diet influences milk microbial ecosystems	4. **Maternal diet** and microbial biodiversity	[1,2,5,10,13,44,45,46]
Moossavi & Azad [11]	2020	Gut Microbes	Human Milk microbiota	Review	Maternal nutrition affects milk microbes		
Pannaraj et al. [33]	2017	JAMA Pediatr	25 Mother-infant dyads	Prospective longitudinal Cohort	Oral/skin microbes contribute to breast milk	5. **Oral and skin microbiota** as sources of colonization	[6,7,47,48,49,50,51,52,53,54,55,56,57,58,59,60,61,62,63,64]
Fernández et al. [4]	2013	Pharm Res	Human Milk microbiome	Review	Skin microbiota part of breast milk ecology		
Moossavi et al. [6]	2019	Cell–Host Microbe	393 Mother–infant dyads	Prospective cohort study (within a birth cohort)	Maternal health influences mammary microbial composition	6. **Breast care** and mammary microbial homeostasis	[4,11,65,66,67,68,69,70,71,72,73,74,75]
Lyons et al. [7]	2020	Nutrients	Human Milk microbiome	Review	Balanced mammary microbiota support infant colonization		
Destoumieux-Garzón [12]	2018	Front Vet Sci	One Health microbiology	Conceptual	Environmental biodiversity supports microbial resilience and health	7. **Environmental exposure**, nature, and physical activity	[14,34,76,77,78,79,80,81,82,83,84,85,86,87,88,89,90,91,92,93,94,95]
Robertson et al. [13]	2019	Trends Microbiol	Early life microbiome	Review	Environment shapes microbiome diversity		
Zijlmans et al. [96]	2015	Psychoneuroendocrinol	56 Mother-infant dyads	Prospective cohort study	Maternal prenatal stress alters infant microbiota	8. **Stress**, neuroendocrine signaling, and microbiota	[13,49,96,97,98,99,100,101]
Donald & Finlay [9]	2023	Nat Rev Immunol	Neuroimmune interactions	Review	Neuroendocrine signals affect microbiota–immune interactions		
Huang et al. [102]	2024	eClinicalMedicine	Infants exposed to treatments	Review	Antibiotics disrupt early microbiota assembly	9. Protecting the microbiota during **medical interventions**	[9,102,103,104,105,106,107,108,109,110,111,112,113,114,115,116,117]
Tamburini et al. [14]	2016	Nat Med	Early life microbiome	Review	Medical exposures alter colonization and microbiota trajectories		
Biagioli et al. [3]	2024	Nutrients	Early-life nutrition	Review	Weaning reshapes microbiome	10. **Complementary feeding** with continued breastfeeding	[14,118,119]
Milani et al. [5]	2017	Microbial Mol biol Rev	Infant gut microbiota	Review	Complementary feeding increases diversity		

**Table 2:** The microbiota-oriented breastfeeding decalogue.

Decalogue Point	Biological Rationale	Microbiota Mechanisms	Health and Care Implications
1. **Breast milk** as a living microbial and signaling ecosystem	Human milk is a dynamic biological fluid conveying microbes, immune cells, and bioactive molecules beyond nutrition	Delivery of milk microbiota, HMOs, immune mediators; promotion of bifidobacteria-dominated ecosystems	Foundational for gut, immune, and neurodevelopment; variability influenced by maternal health and environment
2. **Direct breastfeeding** and microbial transfer	Direct feeding facilitates viable microbial transfer and bidirectional signaling	Retrograde inoculation, reduced oxygen exposure, enhanced survival of maternal microbes	Preferable when feasible; expressed milk retains benefits but may attenuate microbial transfer
3. **Feeding rhythms**, responsiveness, and circadian biology	Early life is sensitive to circadian and hormonal entrainment	Diurnal variation in milk components influences microbial rhythms and immune signaling	Responsive feeding supports physiological regulation; evidence still emerging
4. **Maternal diet** and microbial biodiversity	Maternal nutrition shapes milk metabolites and microbial diversity	Diet-derived substrates act as prebiotics; modulation of milk microbiota composition	Dietary diversity may support bifidobacteria and microbial resilience
5. **Oral and skin microbiota** as sources of colonization	Maternal oral and skin microbiota contribute to early microbial exposure	Transfer via skin contact, oral seeding, and retrograde pathways	Influenced by hygiene practices, mastitis, and environmental factors
6. **Breast care** and mammary microbial homeostasis	Mammary microbiota balance supports lactation and prevents inflammation	Avoidance of dysbiosis through adequate milk drainage and minimal antiseptic disruption	Relevant for mastitis prevention and breastfeeding continuation
7. **Environmental exposure**, nature, and physical activity	Environmental microbial exposure enhances maternal and infant microbial diversity	Immune modulation and enrichment of microbial inputs	Context-dependent; urbanization and lifestyle may limit exposure
8. **Stress**, neuroendocrine signaling, and microbiota	Maternal stress alters hormonal and immune signaling during lactation	Cortisol and oxytocin fluctuations affect milk composition and microbial transfer	Stress management supports both maternal well-being and microbial ecology
9. Protecting the microbiota during **medical interventions**	Medical treatments can disrupt maternal and infant microbiota	Antibiotic-induced dysbiosis; potential mitigation via timing and co-interventions	Risk–benefit assessment essential; microbiota protection is supportive, not overriding
10. **Complementary feeding** with continued breastfeeding	Continued breastfeeding buffers microbiota transitions during weaning	Supports ecological succession and microbial diversification	Gradual, diverse complementary feeding supports tolerance and resilience

## Data Availability

No new data were created or analyzed in this study.

## Abbreviations

The following abbreviations are used in this manuscript.

β-lactam	Beta-lactam antibiotics
HMM	Human Milk Microbiota
HM	Human Milk
HMOs	Human Milk Oligosaccharides
HPA	Hypothalamic–Pituitary–Adrenal (axis)
NICU	Neonatal Intensive Care Unit
NAS	N-acetylserotonin
PPI	Proton Pump Inhibitor
SCFAs	Short-Chain Fatty Acids
TGF-β	Transforming Growth Factor Beta
WHO	World Health Organization
